# Supplementary material for: Modeling glioblastoma heterogeneity as a dynamic network of cell states
Source: Mol Syst Biol. 2021 Sep 16;17(9):e10105. doi: 10.15252/msb.202010105 (PMC8444284; doi:10.15252/msb.202010105)
Supplement: Supplementary file 5 — Source Data for Figure 3 [file MSB-17-e10105-s001.zip › Figure3A_sourcedata/GSEA_3065/nosigdb_state1.GseaPreranked.1622718409535/DOROSZKO_U3180MG_CLUSTER_4.html]

Details for gene set DOROSZKO\_U3180MG\_CLUSTER\_4[GSEA]

|  || Dataset | state1 |
| Phenotype | NoPhenotypeAvailable |
| Upregulated in class | na\_pos |
| GeneSet | DOROSZKO\_U3180MG\_CLUSTER\_4 |
| Enrichment Score (ES) | 0.5563123 |
| Normalized Enrichment Score (NES) | 2.1261342 |
| Nominal p-value | 0.0 |
| FDR q-value | 0.0 |
| FWER p-Value | 0.0 |
Table: GSEA Results Summary

  

Fig 1: Enrichment plot: DOROSZKO\_U3180MG\_CLUSTER\_4      
 Profile of the Running ES Score & Positions of GeneSet Members on the Rank Ordered List

  

| PROBE | GENE SYMBOL | GENE\_TITLE | RANK IN GENE LIST | RANK METRIC SCORE | RUNNING ES | CORE ENRICHMENT || 1 | LGALS1 |  |  | 4 | 0.828 | 0.0443 | Yes |
| 2 | UBE2S |  |  | 19 | 0.634 | 0.0770 | Yes |
| 3 | PTTG1 |  |  | 40 | 0.520 | 0.1030 | Yes |
| 4 | TMSB10 |  |  | 53 | 0.473 | 0.1273 | Yes |
| 5 | PLIN3 |  |  | 75 | 0.410 | 0.1473 | Yes |
| 6 | LDHA |  |  | 80 | 0.394 | 0.1681 | Yes |
| 7 | PSME2 |  |  | 112 | 0.351 | 0.1839 | Yes |
| 8 | ACTN1 |  |  | 230 | 0.284 | 0.1871 | Yes |
| 9 | JPT1 |  |  | 248 | 0.277 | 0.2003 | Yes |
| 10 | GLRX5 |  |  | 255 | 0.276 | 0.2146 | Yes |
| 11 | COPRS |  |  | 369 | 0.235 | 0.2157 | Yes |
| 12 | CCT8 |  |  | 372 | 0.234 | 0.2281 | Yes |
| 13 | RAB13 |  |  | 417 | 0.223 | 0.2356 | Yes |
| 14 | SSNA1 |  |  | 424 | 0.222 | 0.2469 | Yes |
| 15 | MZT1 |  |  | 449 | 0.216 | 0.2561 | Yes |
| 16 | EIF4G2 |  |  | 466 | 0.212 | 0.2659 | Yes |
| 17 | CDC20 |  |  | 469 | 0.212 | 0.2772 | Yes |
| 18 | HDGF |  |  | 512 | 0.203 | 0.2838 | Yes |
| 19 | DAZAP1 |  |  | 530 | 0.199 | 0.2928 | Yes |
| 20 | AURKA |  |  | 550 | 0.196 | 0.3014 | Yes |
| 21 | CCT7 |  |  | 558 | 0.193 | 0.3111 | Yes |
| 22 | TPGS2 |  |  | 569 | 0.190 | 0.3203 | Yes |
| 23 | GTF2A2 |  |  | 581 | 0.189 | 0.3294 | Yes |
| 24 | CCNB1 |  |  | 583 | 0.189 | 0.3395 | Yes |
| 25 | STIP1 |  |  | 623 | 0.182 | 0.3453 | Yes |
| 26 | COX20 |  |  | 629 | 0.182 | 0.3546 | Yes |
| 27 | NDUFAF3 |  |  | 631 | 0.181 | 0.3643 | Yes |
| 28 | ETV4 |  |  | 665 | 0.176 | 0.3704 | Yes |
| 29 | ILF2 |  |  | 729 | 0.167 | 0.3729 | Yes |
| 30 | MAZ |  |  | 768 | 0.162 | 0.3778 | Yes |
| 31 | MORF4L2 |  |  | 789 | 0.158 | 0.3843 | Yes |
| 32 | DCTN3 |  |  | 799 | 0.157 | 0.3918 | Yes |
| 33 | PRR13 |  |  | 814 | 0.155 | 0.3987 | Yes |
| 34 | BZW1 |  |  | 821 | 0.154 | 0.4065 | Yes |
| 35 | ADI1 |  |  | 827 | 0.154 | 0.4142 | Yes |
| 36 | NDUFA2 |  |  | 830 | 0.153 | 0.4223 | Yes |
| 37 | PSMA4 |  |  | 837 | 0.153 | 0.4299 | Yes |
| 38 | CIB1 |  |  | 897 | 0.145 | 0.4317 | Yes |
| 39 | ITGAE |  |  | 917 | 0.143 | 0.4375 | Yes |
| 40 | NUDCD2 |  |  | 929 | 0.142 | 0.4440 | Yes |
| 41 | ANLN |  |  | 941 | 0.141 | 0.4505 | Yes |
| 42 | CEP55 |  |  | 986 | 0.135 | 0.4532 | Yes |
| 43 | MAP1LC3B |  |  | 1027 | 0.130 | 0.4562 | Yes |
| 44 | VBP1 |  |  | 1044 | 0.129 | 0.4615 | Yes |
| 45 | MRPL47 |  |  | 1046 | 0.128 | 0.4683 | Yes |
| 46 | HNRNPH3 |  |  | 1054 | 0.127 | 0.4744 | Yes |
| 47 | RBM42 |  |  | 1108 | 0.121 | 0.4755 | Yes |
| 48 | TROAP |  |  | 1134 | 0.119 | 0.4794 | Yes |
| 49 | UBALD2 |  |  | 1161 | 0.117 | 0.4830 | Yes |
| 50 | TXNDC17 |  |  | 1209 | 0.111 | 0.4842 | Yes |
| 51 | CDKN2D |  |  | 1213 | 0.111 | 0.4899 | Yes |
| 52 | CCNB2 |  |  | 1232 | 0.110 | 0.4939 | Yes |
| 53 | RNF5 |  |  | 1330 | 0.100 | 0.4894 | Yes |
| 54 | ASF1A |  |  | 1355 | 0.099 | 0.4922 | Yes |
| 55 | EMC9 |  |  | 1384 | 0.097 | 0.4946 | Yes |
| 56 | PLK1 |  |  | 1403 | 0.095 | 0.4979 | Yes |
| 57 | RANGAP1 |  |  | 1414 | 0.094 | 0.5019 | Yes |
| 58 | FAM120AOS |  |  | 1471 | 0.091 | 0.5010 | Yes |
| 59 | HNRNPM |  |  | 1476 | 0.091 | 0.5055 | Yes |
| 60 | HMMR |  |  | 1480 | 0.090 | 0.5101 | Yes |
| 61 | CRAT |  |  | 1515 | 0.088 | 0.5113 | Yes |
| 62 | CBX1 |  |  | 1521 | 0.088 | 0.5156 | Yes |
| 63 | NUCKS1 |  |  | 1540 | 0.087 | 0.5184 | Yes |
| 64 | CENPE |  |  | 1545 | 0.086 | 0.5226 | Yes |
| 65 | EZR |  |  | 1549 | 0.086 | 0.5269 | Yes |
| 66 | AAMDC |  |  | 1556 | 0.086 | 0.5310 | Yes |
| 67 | AIMP2 |  |  | 1560 | 0.085 | 0.5353 | Yes |
| 68 | MAP4K4 |  |  | 1579 | 0.084 | 0.5379 | Yes |
| 69 | TIMM9 |  |  | 1590 | 0.083 | 0.5414 | Yes |
| 70 | RUVBL1 |  |  | 1592 | 0.083 | 0.5457 | Yes |
| 71 | SKA2 |  |  | 1599 | 0.082 | 0.5496 | Yes |
| 72 | CDK2AP2 |  |  | 1616 | 0.081 | 0.5523 | Yes |
| 73 | GDI2 |  |  | 1713 | 0.074 | 0.5464 | Yes |
| 74 | SPDL1 |  |  | 1731 | 0.073 | 0.5486 | Yes |
| 75 | CENPA |  |  | 1788 | 0.070 | 0.5467 | Yes |
| 76 | GSPT1 |  |  | 1797 | 0.070 | 0.5496 | Yes |
| 77 | SYNCRIP |  |  | 1817 | 0.069 | 0.5514 | Yes |
| 78 | SAPCD2 |  |  | 1829 | 0.068 | 0.5539 | Yes |
| 79 | KIF18A |  |  | 1842 | 0.067 | 0.5563 | Yes |
| 80 | DLGAP5 |  |  | 2028 | 0.058 | 0.5404 | No |
| 81 | ECI2 |  |  | 2031 | 0.058 | 0.5433 | No |
| 82 | RUVBL2 |  |  | 2064 | 0.057 | 0.5431 | No |
| 83 | PAIP1 |  |  | 2090 | 0.055 | 0.5435 | No |
| 84 | NCAPD2 |  |  | 2121 | 0.054 | 0.5433 | No |
| 85 | MDH1 |  |  | 2144 | 0.053 | 0.5439 | No |
| 86 | TMEM256 |  |  | 2147 | 0.053 | 0.5466 | No |
| 87 | ECT2 |  |  | 2150 | 0.053 | 0.5492 | No |
| 88 | CDCA8 |  |  | 2166 | 0.052 | 0.5505 | No |
| 89 | USP13 |  |  | 2173 | 0.052 | 0.5527 | No |
| 90 | NDE1 |  |  | 2284 | 0.048 | 0.5440 | No |
| 91 | PRR11 |  |  | 2329 | 0.046 | 0.5419 | No |
| 92 | LMO7 |  |  | 2336 | 0.046 | 0.5438 | No |
| 93 | CDC25B |  |  | 2370 | 0.044 | 0.5428 | No |
| 94 | GPSM2 |  |  | 2477 | 0.040 | 0.5340 | No |
| 95 | FAM83D |  |  | 2491 | 0.040 | 0.5348 | No |
| 96 | KNSTRN |  |  | 2515 | 0.039 | 0.5346 | No |
| 97 | EIF4E |  |  | 2517 | 0.039 | 0.5366 | No |
| 98 | DDAH2 |  |  | 2535 | 0.038 | 0.5369 | No |
| 99 | SPAG5 |  |  | 2566 | 0.038 | 0.5358 | No |
| 100 | CEP70 |  |  | 2601 | 0.036 | 0.5343 | No |
| 101 | TNFAIP8L1 |  |  | 2612 | 0.036 | 0.5352 | No |
| 102 | EEF1AKMT2 |  |  | 2620 | 0.036 | 0.5364 | No |
| 103 | RHNO1 |  |  | 2700 | 0.033 | 0.5301 | No |
| 104 | SEPHS1 |  |  | 2755 | 0.031 | 0.5262 | No |
| 105 | ANP32A |  |  | 2765 | 0.031 | 0.5269 | No |
| 106 | NEK2 |  |  | 2834 | 0.030 | 0.5215 | No |
| 107 | CALM3 |  |  | 2882 | 0.028 | 0.5182 | No |
| 108 | BUB1 |  |  | 2888 | 0.028 | 0.5192 | No |
| 109 | KIF20A |  |  | 2897 | 0.028 | 0.5199 | No |
| 110 | RNF26 |  |  | 3049 | 0.024 | 0.5056 | No |
| 111 | RANGRF |  |  | 3091 | 0.023 | 0.5026 | No |
| 112 | PPP1CC |  |  | 3152 | 0.021 | 0.4976 | No |
| 113 | HNRNPA1P48 |  |  | 3156 | 0.021 | 0.4984 | No |
| 114 | ODF2 |  |  | 3157 | 0.021 | 0.4995 | No |
| 115 | CCDC18 |  |  | 3447 | 0.015 | 0.4706 | No |
| 116 | SORD |  |  | 3535 | 0.013 | 0.4623 | No |
| 117 | AC026401.3 |  |  | 3542 | 0.013 | 0.4624 | No |
| 118 | KIAA0586 |  |  | 3569 | 0.012 | 0.4604 | No |
| 119 | TTK |  |  | 3724 | 0.009 | 0.4451 | No |
| 120 | RAB11A |  |  | 3749 | 0.009 | 0.4431 | No |
| 121 | KHDRBS1 |  |  | 3753 | 0.009 | 0.4433 | No |
| 122 | PPP1R35 |  |  | 3774 | 0.009 | 0.4417 | No |
| 123 | TMEM19 |  |  | 3843 | 0.007 | 0.4351 | No |
| 124 | FKBP2 |  |  | 3905 | 0.006 | 0.4291 | No |
| 125 | REEP4 |  |  | 4020 | 0.004 | 0.4176 | No |
| 126 | NUP37 |  |  | 4278 | -0.001 | 0.3912 | No |
| 127 | SRRT |  |  | 4303 | -0.001 | 0.3887 | No |
| 128 | PRMT5 |  |  | 4376 | -0.002 | 0.3815 | No |
| 129 | NUDT11 |  |  | 4390 | -0.003 | 0.3803 | No |
| 130 | G2E3 |  |  | 4397 | -0.003 | 0.3798 | No |
| 131 | SNAPC1 |  |  | 4657 | -0.007 | 0.3535 | No |
| 132 | CFAP36 |  |  | 4761 | -0.008 | 0.3433 | No |
| 133 | PCBD2 |  |  | 4803 | -0.009 | 0.3396 | No |
| 134 | GAS2L3 |  |  | 4918 | -0.011 | 0.3285 | No |
| 135 | PPP2R5C |  |  | 4964 | -0.012 | 0.3245 | No |
| 136 | KATNA1 |  |  | 4983 | -0.012 | 0.3233 | No |
| 137 | SEC22C |  |  | 5077 | -0.014 | 0.3145 | No |
| 138 | KIF20B |  |  | 5169 | -0.015 | 0.3059 | No |
| 139 | IMMP1L |  |  | 5239 | -0.016 | 0.2997 | No |
| 140 | KIF14 |  |  | 5262 | -0.016 | 0.2983 | No |
| 141 | HP1BP3 |  |  | 5264 | -0.016 | 0.2990 | No |
| 142 | SPA17 |  |  | 5521 | -0.020 | 0.2738 | No |
| 143 | ARPP19 |  |  | 5555 | -0.021 | 0.2715 | No |
| 144 | SMIM14 |  |  | 6092 | -0.031 | 0.2180 | No |
| 145 | SUN2 |  |  | 6172 | -0.032 | 0.2116 | No |
| 146 | KIF11 |  |  | 6477 | -0.038 | 0.1824 | No |
| 147 | FUBP1 |  |  | 7038 | -0.051 | 0.1275 | No |
| 148 | RBMX |  |  | 7133 | -0.054 | 0.1207 | No |
| 149 | IFT22 |  |  | 7211 | -0.056 | 0.1158 | No |
| 150 | CKAP5 |  |  | 7381 | -0.060 | 0.1016 | No |
| 151 | EMP3 |  |  | 7404 | -0.061 | 0.1026 | No |
| 152 | TMEM160 |  |  | 7653 | -0.068 | 0.0807 | No |
| 153 | CDKN2C |  |  | 7660 | -0.068 | 0.0838 | No |
| 154 | TMED5 |  |  | 7679 | -0.069 | 0.0857 | No |
| 155 | DCP2 |  |  | 7913 | -0.078 | 0.0659 | No |
| 156 | CNIH4 |  |  | 8025 | -0.082 | 0.0589 | No |
| 157 | ZNF106 |  |  | 8417 | -0.100 | 0.0240 | No |
| 158 | HSD17B11 |  |  | 8769 | -0.123 | -0.0055 | No |
| 159 | SDCBP |  |  | 8790 | -0.124 | -0.0009 | No |
| 160 | TXNDC12 |  |  | 8811 | -0.125 | 0.0039 | No |
| 161 | ARL6IP1 |  |  | 9038 | -0.146 | -0.0115 | No |
| 162 | LBR |  |  | 9264 | -0.174 | -0.0253 | No |
| 163 | PIF1 |  |  | 9278 | -0.176 | -0.0171 | No |
| 164 | MEX3A |  |  | 9384 | -0.197 | -0.0173 | No |
| 165 | HSPA13 |  |  | 9428 | -0.206 | -0.0106 | No |
| 166 | SUGP2 |  |  | 9618 | -0.265 | -0.0157 | No |
| 167 | IDH1 |  |  | 9737 | -0.345 | -0.0092 | No |
| 168 | PDGFRA |  |  | 9806 | -0.440 | 0.0075 | No |
Table: GSEA details [plain text format]

  

Fig 2: DOROSZKO\_U3180MG\_CLUSTER\_4: Random ES distribution      
 Gene set null distribution of ES for **DOROSZKO\_U3180MG\_CLUSTER\_4**
